# Withholding vs. Continuing Angiotensin-Converting Enzyme Inhibitors or Angiotensin Receptor Blockers Before Non-cardiac Surgery in Older Patients: Study Protocol for a Multicenter Randomized Controlled Trial

**DOI:** 10.3389/fmed.2021.654700

**Published:** 2021-03-30

**Authors:** Yu-fan Yang, Ya-juan Zhu, Yu-qin Long, Hua-yue Liu, Xi-sheng Shan, Xiao-mei Feng, Ke Peng, Fu-hai Ji

**Affiliations:** ^1^Department of Anesthesiology, First Affiliated Hospital of Soochow University, Suzhou, China; ^2^Department of Anesthesiology, University of Utah Health, Salt Lake City, UT, United States

**Keywords:** angiotensin-converting enzyme inhibitors, angiotensin receptor blockers, older patients, non-cardiac surgery, hypotension, post-operative outcomes

## Abstract

**Background:** Older hypertensive adults are at increased risk for postoperative morbidity and mortality. As first line antihypertensive drug therapy, angiotensin-converting enzyme inhibitors (ACEIs) or angiotensin receptor blockers (ARBs) have many beneficial effects. However, the use of ACEIs/ARBs in the perioperative period remains controversial. This study aims to determine the effects of withholding vs. continuing ACEIs/ARBs before non-cardiac surgery on perioperative hypotension and postoperative outcomes in older patients.

**Methods:** In this multicenter, randomized, double-blind, placebo-controlled trial, a total of 2036 patients aged 60–80 years undergoing non-cardiac surgical procedures will be randomly assigned, in a 1:1 ratio, to receive oral ACEIs/ARBs (the ACEIs/ARBs continued group) or inactive placebos (the ACEIs/ARBs withheld group) on the morning of surgery. For both groups, the ACEIs/ARBs will be continued from the first postoperative day. The primary outcome measure is the incidence of perioperative hypotensive events, defined as mean blood pressure (MBP) < 65 mmHg or ≥30% reduction in MBP from baseline during surgery and in a post-anesthesia care unit. The secondary outcomes include duration of perioperative hypotension, intraoperative use of fluids and vasopressors, hypotensive events within postoperative 3 days, and perioperative neurocognitive disorders, major adverse cardiocerebral events (a composite outcome of stroke, coma, myocardial infarction, heart block, and cardiac arrest), and mortality within 30 days after surgery.

**Discussion:** The results of this trial will offer an evidence-based perioperative ACEIs/ARBs therapy for older hypertensive adults undergoing non-cardiac surgery.

**Study Registration:** This study is approved by the Medical Ethics Committee of The First Affiliated Hospital of Soochow University (Approval No. 2020-077-1) and by the institutional ethics review board of each participating center. This protocol is registered at the Chinese Clinical Trials Registry (ChiCTR2000039376).

## Introduction

The era of geriatric surgery has arrived (1, 2). With a rapidly aging global population, increasing numbers of surgical operations are being performed on older adults. While several surgical risk factors increase with age, increasing age itself independently predicts postoperative morbidity and mortality (3). Older patients who undergo surgical procedures present great challenges in the perioperative management. To improve the outcomes after geriatric surgery, intensive efforts are required, including careful preoperative risk assessment, treatment for comorbidities, optimal surgical techniques and anesthesia management, and implementation of enhanced recovery after surgery.

Hypertension is a leading preoperative risk factor among patients of all age groups and dominates in the group aged 50 years and older (3–5). Preoperative hypertension increases the risk of surgical bleeding, stroke, and cardiovascular complications. Tight blood pressure control improves outcome. According to the 2017 American College of Cardiology/American Heart Association Task Force on Clinical Practice Guidelines, blood pressure should be <130/80 mmHg during an antihypertensive drug therapy (6). As first line antihypertensive treatment, angiotensin-converting enzyme inhibitors (ACEIs) or angiotensin receptor blockers (ARBs) produce arterial vasodilatation and reduce blood pressure through the inhibition of the renin–angiotensin aldosterone system (4). Moreover, the use of ACEIs/ARBs exerts many beneficial effects, including improved cardiovascular and renal outcomes in patients with diabetes, decreased mortality in patients with acute kidney injury, and improvement in symptoms and survival in patients with heart failure with reduced ejection fraction (7–10).

Nevertheless, the current evidence does not provide a definitive answer regarding whether ACEIs/ARBs should be continued or withheld in the perioperative setting. While some studies showed that continuation of ACEIs/ARBs was associated with an increased risk of intraoperative hypotension (11–15), other studies argued that different ACEIs/ARBs management strategies did not affect perioperative hemodynamic stability or postoperative outcomes (16–19). To date, there is no well-designed randomized controlled trial with adequate power to assess the perioperative risks and benefits of ACEIs/ARBs.

Therefore, the present study aims to determine the effects of withholding vs. continuing ACEIs/ARBs before non-cardiac surgery in older hypertensive adults. The primary outcome measure of this study is the incidence of perioperative hypotension that occurs during surgery and in a post-anesthesia care unit (PACU). The secondary outcome measures include duration of perioperative hypotension, intraoperative use of fluids and vasopressors, and hypotensive events within postoperative 3 days, as well as neurocognitive function, cardiocerebral outcomes, and mortality within 30 days after surgery. We will mainly test the hypothesis that the withholding of ACEI on the day of surgery will decrease the number of perioperative hypotensive events in patients undergoing non-cardiac surgery.

## Materials and Methods

### Study Design

This study is a multicenter, randomized, double-blind, placebo-controlled trial conducted at six tertiary hospitals in China. The leading site is The First Affiliated Hospital of Soochow University, and the participating sites are Zhongshan Hospital Fudan University, Fudan University Shanghai Cancer Center, Shanghai General Hospital, The First Affiliated Hospital of Zhengzhou University, and The First Affiliated Hospital of Anhui Medical University.

### Ethics and Dissemination

The study protocol is approved by the Medical Ethics Committee of The First Affiliated Hospital of Soochow University (Approval No. 2020-077-1) and by the institutional ethics review board of each participating center. This study is registered at the Chinese Clinical Trials Registry on October 25, 2020 (http://www.chictr.org.cn, identifier: ChiCTR2000039376), before the enrollment of the first subject. This study follows the Declaration of Helsinki and the International Conference on Harmonization guidelines for Good Clinical Practice. This report follows the Standard Protocol Items: Recommendations for Interventional Trials (SPIRIT) statement (20).

### Participants

**Figure 1:**
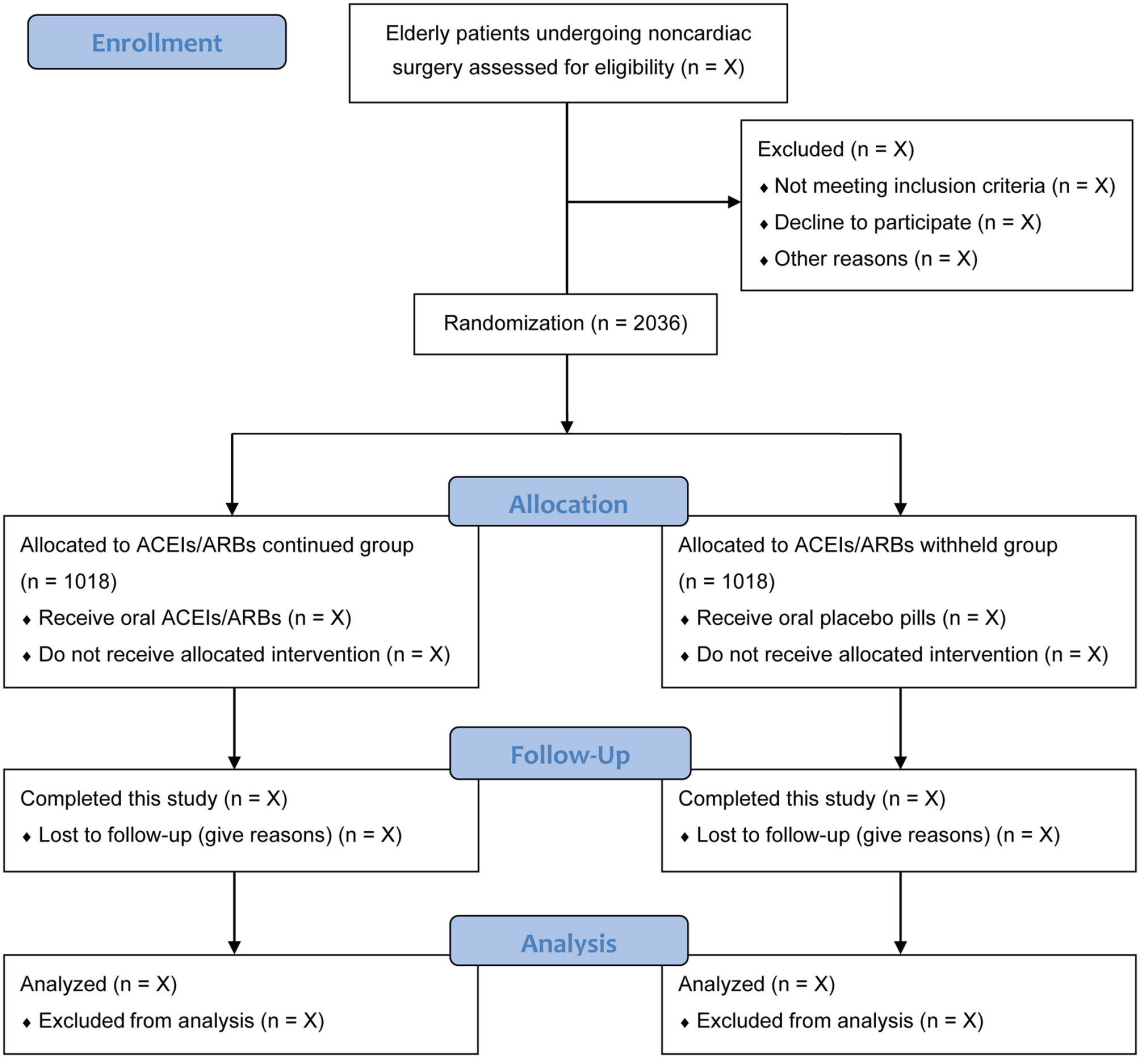
Flow chart of participants. ACEIs/ARBs, angiotensin-converting enzyme inhibitors or angiotensin receptor blockers.

At each study center, an independent preoperative investigator who is not involved in patient care or data management will screen patients' admission records to identify potentially qualified participants. Patients who meet the eligibility criteria and provide their written informed consent will be included in this study. According to the sample size estimation, a total of 2,036 eligible patients who plan to undergo non-cardiac surgical procedures will be randomly assigned, in a 1:1 ratio, to an ACEIs/ARBs continued group or an ACEIs/ARBs withheld group. Patients can withdraw their consent at any time during this study. The patient recruitment started on November 1, 2020. The expected date for completion of recruitment is December 2023. The flow chart of participants is shown in Figure 1.

### Inclusion and Exclusion Criteria

Patients are eligible for this trial if they are 60–80 years old with ASA physical status II or III, receiving regular ACEIs or ARBs therapy for hypertension for more than 2 weeks, who are scheduled for non-cardiac surgery under general anesthesia.

The exclusion criteria are as follows: (1) unplanned or emergent surgery, (2) spinal and/or epidural anesthesia, (3) ACEIs/ARBs use only in the evening, intermittent ACEIs/ARBs use, or concomitant use of ACEIs and ARBs, (4) uncontrolled hypertension defined as systolic blood pressure (SBP) ≥ 180 mmHg or diastolic blood pressure (DBP) ≥ 110 mmHg, (5) baseline mean blood pressure (MBP) ≤ 65 mmHg, (6) intravenous use of vasoactive agents, (7) severe cardiopulmonary or cerebrovascular disease, (8) liver or kidney dysfunction (Child-Pugh grade ≥ B, creatinine ≥ 200 μmol/L), or (9) rejection of participation.

### Randomization and Blinding

An independent biostatistician who is not involved in the following study or data management generated a randomization sequence with a 1:1 ratio, permuted block sizes of 2 and 4, and stratification according to study center. Randomization is implemented by using a web-based central randomization system, and allocation concealment is ensured by password protection.

According to the random codes, an independent research nurse will prepare the study medications (either ACEIs/ARBs or inactive placebos) in identical capsules, which were kept in bags labeled with the study numbers and “Antihypertensive agent.” Subjects who meet the eligibility criteria will be randomly assigned to either an ACEIs/ARBs continued group or an ACEIs/ARBs withheld group. The participants, anesthesiologists, surgeons, investigators responsible for data collection, and other healthcare providers will remain masked to treatment allocation until the completion of final analysis.

In case of a severe adverse event considered to be related to study interventions, the attending anesthesiologists or physicians should notify the principal investigator to decide whether unmasking of treatment allocation is needed, and such an event will be documented by an independent data monitoring committee (DMC).

### Study Interventions and Anesthesia

The schedule of enrollment, interventions, and assessments in accordance with the SPIRIT 2013 statement is shown in Table 1. The baseline blood pressure is obtained by independent nursing personnel when patients are comfortably seated in surgical wards before the day of surgery. According to the web-based centralized randomization list, patients will take the study medications (either the ACEIs/ARBs or placebo pills) orally on the morning of surgery. The time of study medication will be documented.

**Table 1:** Schedule of enrollment, interventions, and assessments.

		**Study period**							
	**Enrollment**	**Allocation**	**Post-allocation**						**Close-out**
**Timepoint**	**Preanesthetic visit**	**Prior to surgery**	**During surgery**	**PACU**	**POD 1**	**POD 2**	**POD 3**	**Hospital discharge**	**POD 30**
**Enrollment**									
Inclusion criteria	×								
Exclusion criteria	×								
Informed consent	×								
Demographics	×								
Comorbidities	×								
Randomization	×								
Allocation		×							
**Interventions**									
ACEIs/ARBs continued		×							
ACEIs/ARBs withheld		×							
**Assessments**									
Baseline blood pressure		×							
Hypotension events			×	×	×	×	×		
Other hemodynamic events			×	×	×	×	×		
Fluids and vasoactive agents			×	×					
Intraoperative adverse events			×						
Pain, analgesic use, PONV				×	×	×	×		
ICU admission								×	
Length of postoperative stay								×	
PND, MACE, and mortality				×	×	×	×	×	×

According to SPIRIT 2013 statement of defining standard protocol items for clinical trials.

ACEI, angiotensin converting enzyme inhibitor; ARB, angiotensin receptor antagonist; PONV, postoperative nausea and vomiting; PND, neurocognitive disorders; MACE, major adverse cardiocerebral events; POD, postoperative day.

In the operating room, patients will receive a standard monitoring including heart rate (HR), non-invasive blood pressure, and pulse oximetry. Induction of general anesthesia will be performed with sufentanil 0.2–0.4 μg/kg and etomidate 0.2–0.3 mg/kg. Tracheal intubation will be facilitated with rocuronium 0.6 mg/kg or cisatracurium 0.2 mg/kg. Mechanical ventilation will be performed with a tidal volume of 8–10 ml/kg, frequency of 12–18 breaths/min, adjusted to maintain end-tidal CO_2_ values within 30–40 mmHg. General anesthesia will be maintained with propofol infusion titrated to bispectral index (BIS) values within 40–60, or with sevoflurane inhalation titrated to end-tidal minimal alveolar concentration within 0.8–1.3. To provide adequate intraoperative analgesia and neuromuscular blockade, additional doses of sufentanil and rocuronium (or cisatracurium) can be administered. Body temperature will be maintained at 36–37°C with a nasopharyngeal probe by using a warming blanket and/or an infusion heating device. Other perioperative management will be left to the discretion of the attending anesthesiologists according to the routine practice of each study center. After tracheal extubation, patients will be transferred to the PACU. When a modified Aldrete score ≥ 9 is achieved, patients will be discharged from the PACU to the surgical wards.

For both groups, the use of ACEIs/ARBs will be continued in the morning of the first post-operative day. If a perioperative hypertensive event occurs during the study period, patients will receive antihypertensive treatments based on the institutional clinical practice.

### Outcome Measures

The primary outcome measure of this trial is the incidence of perioperative hypotensive events, defined as MBP <65 mmHg or ≥30% reduction in MBP from baseline for which an intervention is needed (including intravenous fluids infusion and/or use of vasopressors), which occur during surgery and in the PACU.

The secondary outcome measures are: (1) duration of perioperative hypotension, (2) intraoperative use of fluids and vasopressors, (3) postoperative hypotensive events in the surgical wards within 3 days after surgery, and (4) perioperative neurocognitive disorders (PND), major adverse cardiocerebral events (MACEs, a composite outcome of permanent or transient stroke, coma, myocardial infarction, heart block, and cardiac arrest), and mortality within 30 days after surgery. MACE will be assessed as a composite outcome, and each complication will also be assessed individually, according to the definitions in Table 2.

**Table 2:** Definitions of postoperative complications.

**Event**	**Definition**
PND	Indicates perioperative neurocognitive disorders assessed using the CAM-ICU until ICU discharge or using the CAM in the surgical wards and at 30 days after surgery.
MACEs	Indicates a composite outcome of permanent or transient stroke, coma, myocardial infarction, heart block, and cardiac arrest.
Stroke	Indicates a postoperative stroke (i.e., any confirmed neurological deficit of abrupt onset caused by a disturbance in blood supply to the brain).
Coma	Indicates a new postoperative coma that persists for at least 24 h secondary to anoxic/ischemic and/or metabolic encephalopathy, thromboembolic event or cerebral bleed.
Myocardial infarction	Indicates a myocardial infarction event documented by at least one of the following criteria: 1. evolutionary ST-segment elevations; 2. development of new Q-waves in two or more contiguous ECG leads; 3. new or presumably new left bundle branch block pattern on the ECG; 4. cardiac Troponin I > 0.05 ng/ml.
Heart block	Indicates a new heart block requiring the implantation of a permanent pacemaker of any type prior to discharge.
Cardiac arrest	Indicates an acute cardiac arrest documented by one of the following: 1. ventricular fibrillation; 2. rapid ventricular tachycardia with hemodynamic instability; 3. asystole.

*PND, perioperative neurocognitive disorders; MACEs, major adverse cardiocerebral events; CAM, confusion assessment method; ICU, intensive care unit; ECG, electrocardiogram*.

The exploratory outcomes are as follows: (1) the incidence of hypertensive events (MBP > 110 mmHg or ≥ 30% increase in MBP from baseline) during surgery, in the PACU, and within 3 postoperative days, (2) tachycardia or bradycardia events defined as HR > 100 beats/min or HR <50 beats/min, (3) intraoperative adverse events (arrythmia, hypoxia, allergic reaction, major bleeding, hyperthermia, hypothermia, oliguria, acid-base disturbances, electrolyte disorders, lactic acidosis, and cardiac arrest), (4) Ramsay sedation scores in the PACU, (5) numerical rating scale scores for pain, analgesic consumption, and postoperative nausea and vomiting (PONV) in the PACU and in the wards within three post-operative days, (6) time to extubation, (7) intensive care unit (ICU) admission, and (8) length of postoperative hospital stay.

### Data Collection

Patients' demographics, baseline characteristics, and intraoperative data will be collected in the Case Report Forms by independent investigators. Trained postoperative assessors who are unaware of group allocation will collect postoperative in-hospital data by ward visits and 30-day follow-up data *via* telephone. The lead investigator of each study center is responsible for the accuracy and completeness of data. Site visits will be carried out by two investigators for source data verification during the implementation of this study. After de-identification, all data will be stored electronically in a web-based database (https://www.91trial.com/) and monitored by the independent data monitoring committee.

### Sample Size Estimation

The sample size calculation was performed using the PASS software (version 11.0.7, NCSS, LCC, Kaysville, UT, USA). Based on an international prospective cohort study of 4802 patients undergoing non-cardiac surgery, the incidence of intraoperative hypotensive events was 28.6% in the ACEIs/ARBs continued group (15). We hypothesize that the suspension of ACEIs/ARBs before surgery would reduce the incidence of hypotension by 20%, that is, to a hypotension rate of 22.88%. To detect such a difference with α = 0.05 and power = 80%, 916 patients per group are needed. We estimate a drop-out rate of 10%, and thus 1,018 patients will be enrolled in each group.

### Statistical Analysis

Data distribution and normality will be assessed with Shapiro-Wilk test. Continuous variables will be presented as mean ± standard deviation or median (interquartile ranges), depending on their distribution. Categorical variables will be presented as number (percentages). All analyses will follow the intention-to-treat principle, which includes all participants after randomization and excludes the patients who drop out of the study due to withdrawal of informed consent or cancellation of surgery. The per-protocol analysis will be carried out as a sensitivity analysis, which includes all participants with planned interventions and minimal protocol violation. As we expect that the missing data will be uncommon in our dataset, there will be no plan for imputation of missing data.

The primary outcome of perioperative hypotension occurrence will be assessed using multivariate logistic regression adjusting for the following baseline covariates: age, body mass index, ASA status, hypertension grade, previous myocardial infarction, atrial fibrillation, diabetes mellitus, hemoglobin value, use of diuretics, and trial site. The odds ratio with 95% confidence intervals will be reported.

In addition, subgroup analyses for the primary outcome will be conducted to explore whether the effects of study interventions will vary, according to six variables: age, hypertension grade, diabetes mellitus, type of surgery, duration of surgery, and trial site. The interaction analysis of effects across the subgroups will be performed using a test of treatment-by-covariate interaction on a logistic regression model.

For the secondary outcomes, analysis will be conducted using generalized linear model for continuous variables or using multivariate logistic regression for binary variables, adjusting for the above-mentioned baseline covariates. The Benjamini-Hochberg procedure will be used to control the false-discovery rate for multiple comparisons. The *P*-values before and after correction will be presented. For the exploratory outcomes, only descriptive statistics will be applied, without multiple testing or statistical inference.

It is expected that the risk of the study interventions will not be significantly higher compared to the standard medical practice, and there is no ethically risk associated with the primary outcome assessment. Hence, interim analysis will not be performed. All analyses will be done using the SAS software (version 9.4, SAS Institute Inc., Cary, NC, USA) and R statistical software (version 3.6.0, R Foundation for Statistical Computing, Vienna, Austria), with a two-sided *P*-value < 0.05 indicating a statistically significant difference.

## Discussion

In this multicenter, randomized, double-blind, placebo-controlled trial of older hypertensive adults undergoing noncardiac surgery, the primary objective is to evaluate the effects of withholding vs. continuing ACEIs/ARBs before surgery on the incidence of perioperative hypotensive events which occur during surgery and in the PACU. In addition, we will analyze the duration of perioperative hypotension, intraoperative use of fluids and vasopressors, hypotensive events within postoperative 3 days, and the rates of PND, MACEs, and mortality within 30 days after surgery. This trial will follow the Consolidated Standards of Reporting Trials (CONSORT) guideline (21).

Hypotension is significantly associated with postoperative myocardial infarction, acute kidney injury, and death (22–25). In addition, the duration of hypotension was significantly associated with postoperative stroke for patients undergoing non-cardiac and non-neurosurgical procedures (26). Randomized studies also confirmed that interventions targeting an optimal blood pressure reduced the risk of organ dysfunction after major non-cardiac surgery (27, 28). In a recent study of 955 patients with coronary artery disease and mean age of 69.7 years undergoing major non-cardiac surgery, perioperative hypotension independently predicts cardiovascular events (myocardial infarction or cardiovascular death) within 30 days after surgery (29). Older hypertensive patients have impaired autoregulation of blood flow in the vital organs (such as heart, brain, and kidneys) and are at increased postoperative risk, so perioperative blood pressure management is extremely important. Strategies that limit perioperative hypotension contribute to the improvement of postoperative outcomes.

There are no firm recommendations on the use of ACEIs/ARBs in the perioperative period, because high level of evidence such as a randomized controlled trial with a large sample size is lacking. Previous observational studies suggested that withholding ACEIs/ARBs was associated with a lower incidence of intraoperative hypotension and a reduced risk of 30-day vascular events and all-cause mortality (14, 15). However, a recent cohort study did not show an association between ACEIs/ARBs use and hypotension after general anesthesia induction in non-cardiac surgery (18). In addition, an open-label randomized trial suggested that preoperative ACEIs/ARBs management strategies did not affect vasoactive agent use or postoperative outcomes for cardiac surgical patients (19). In a recent meta-analysis of 5 randomized controlled trials and 4 cohort studies in non-cardiac surgery, continuing ACEIs/ARBs was associated with an increased incidence of intraoperative hypotensive events, but an association between ACEIs/ARBs use and major cardiac events or mortality was not found (30). Of note, the associated increase in hypotensive events in the ACEIs/ARBs continued group was mainly based on observational studies. In fact, only 3 randomized trials with a total of 75 patients reported intraoperative hypotension in this meta-analysis (11, 13, 31). A significant limitation of observational data is that the apparent associations are often confounded by multiple factors that can be related to both exposures and outcomes, making it hard to interpret the data and offering a relatively low level of evidence for clinical practice.

To our knowledge, this is the first multicenter, randomized, double-blind, placebo-controlled trial with an adequate power to assess the impact of ACEIs/ARBs management strategies on the occurrence of perioperative hypotension and major complications in older patients undergoing non-cardiac surgery. The second strength of the present trial is that, to increase the precision of statistical estimates, the primary analysis of perioperative hypotension will be adjusted using a multivariate logistic regression model for a number of baseline covariates, based on the knowledge of the association between each variable and patient outcome. Next, to explore whether the effects of study interventions will vary for several variables, subgroup analyses for the primary outcome will be performed using a logistic regression model.

There are also some limitations. First, the primary outcome of this trial is the incidence of perioperative hypotensive events, and we designate postoperative cardiovascular and cognitive complications and mortality within 30 days after surgery as the secondary outcomes. This study would be even more impactful if a composite of cerebral and cardiovascular complications is designated as the primary outcome. However, that would increase the sample size to more than 5,400 patients in order to detect a 20% decrease in the incidence of a composite of stroke, myocardial injury, or death (α = 0.05, power = 80%), based on the previous study (15). Next, we include older adults with ASA physical status II and III, and thus the effects of withholding or continuing ACEIs/ARBs strategies on patients who are sicker (i.e., ASA status IV) need to be assessed in future studies. Last, we exclude very elderly patients who are > 80 years old in this study, because we believe that further investigations on this extremely vulnerable patient population should be carried out carefully, on the basis of the results of our present study.

In conclusion, this trial is designed to determine the effects of the ACEIs/ARBs withheld strategy, as compared to the ACEIs/ARBs continued approach, on perioperative hypotension and postoperative outcomes in older patients who undergo non-cardiac surgical procedures. The implementation and reporting of this trial will be in accordance with the CONSORT guideline (21). We expect that our results will offer an evidence-based perioperative ACEIs/ARBs therapy for older hypertensive adults undergoing non-cardiac surgery.

## Ethics Statement

The studies involving human participants were reviewed and approved by Medical Ethics Committee of The First Affiliated Hospital of Soochow University. The patients/participants provided their written informed consent to participate in this study.

## Author Contributions

All authors made substantial contributions to the design of this study, data acquisition and interpretation, statistical plan, drafting the manuscript, or revising the manuscript critically. All authors agreed to be accountable for all aspects of the work and gave their final approval of this version to be published.

## Conflict of Interest

The authors declare that the research was conducted in the absence of any commercial or financial relationships that could be construed as a potential conflict of interest.
